# Modeling livestock population structure: a geospatial database for Ontario swine farms

**DOI:** 10.1186/s12917-018-1362-y

**Published:** 2018-01-30

**Authors:** Salah Uddin Khan, Terri L. O’Sullivan, Zvonimir Poljak, Janet Alsop, Amy L. Greer

**Affiliations:** 1 Department of Population Medicine, Ontario Veterinary College, University of Guelph, Guelph, ON Canada; 2 Ontario Ministry of Agriculture, Food and Rural Affairs, Guelph, ON Canada

**Keywords:** Synthetic population, Swine, Pigs, Farms, Ontario, Infectious disease transmission model

## Abstract

**Background:**

Infectious diseases in farmed animals have economic, social, and health consequences. Foreign animal diseases (FAD) of swine are of significant concern. Mathematical and simulation models are often used to simulate FAD outbreaks and best practices for control. However, simulation outcomes are sensitive to the population structure used. Within Canada, access to individual swine farm population data with which to parameterize models is a challenge because of privacy concerns. Our objective was to develop a methodology to model the farmed swine population in Ontario, Canada that could represent the existing population structure and improve the efficacy of simulation models.

**Results:**

We developed a swine population model based on the factors such as facilities supporting farm infrastructure, land availability, zoning and local regulations, and natural geographic barriers that could affect swine farming in Ontario. Assigned farm locations were equal to the swine farm density described in the 2011 Canadian Census of Agriculture. Farms were then randomly assigned to farm types proportional to the existing swine herd types. We compared the swine population models with a known database of swine farm locations in Ontario and found that the modeled population was representative of farm locations with a high accuracy (AUC: 0.91, Standard deviation: 0.02) suggesting that our algorithm generated a reasonable approximation of farm locations in Ontario.

**Conclusion:**

In the absence of a readily accessible dataset providing details of the relative locations of swine farms in Ontario, development of a model livestock population that captures key characteristics of the true population structure while protecting privacy concerns is an important methodological advancement. This methodology will be useful for individuals interested in modeling the spread of pathogens between farms across a landscape and using these models to evaluate disease control strategies.

**Electronic supplementary material:**

The online version of this article (10.1186/s12917-018-1362-y) contains supplementary material, which is available to authorized users.

## Background

The Canadian swine industry has gone through a major transformation in the last century. The number of pig farms has decreased from 8.1 in 1921 to 0.6 per 100 inhabitants in 2011 [1], but the average number of pigs raised per farm has continued to grow in Canada. In 2011, the swine industry was the fourth largest agricultural industry (CAD 3.9 billion value) in Canada after canola, dairy products and beef cattle [1]. During the same year, Canada exported more than one million tons of pork valued at $2.9 billion to more than 80 countries and was ranked as the fifth largest pork exporter in the world [2]. Ontario is considered to be the second largest swine producing province in Canada with 24.4% of all pigs and the majority are in southern Ontario [3]. It is hypothesized that areas with a high density of farms are at greater risk for infectious disease introduction and spread [4, 5].

Infectious diseases in farmed animals have economic, social, and animal health consequences as well a possible human health consequences [6]. Foreign animal diseases (FAD) of swine are of significant concern in terms of animal health and welfare and also due to the anticipated economic losses that would arise as a result of a FAD incursion in Ontario [2]. Because FAD introductions are rare events especially in more developed countries with rigorous importation regulations on animals and animal products, mathematical and epidemiological modeling are often used to simulate the extent of such outbreaks and best practices for control using what are considered to be the “most likely” scenarios [7].

Disease modeling provides a cost-effective approach for researchers to develop an understanding of the determinants of disease spread within a population and factors contributing to effective disease control [8]. Host-pathogen relationships frequently demonstrate non-linear trends. Such complex relationships are often best described by computer simulation models [8]. This methodology has contributed to our understanding of disease dynamics and how best to intervene in order to prevent the spread of an infectious disease [8, 9]. However, simulation outcomes are sensitive to the geographic location of livestock enterprises, the number of animals present at each location, and the frequency of both direct and indirect movement events between livestock enterprise locations [10, 11]. In Canada, access to individual swine farm population data with which to parameterize models is limited because of privacy concerns. Authorities responsible for disease outbreak response such as Canadian Food Inspection Agency (CFIA) and Ontario Ministry of Agriculture, Food and Rural Affairs (OMAFRA) do not maintain a livestock population database of animals susceptible to FAD or other reportable diseases. Rather, they rely on premise and animal identification databases maintained by the provincial or territorial governments and/or by livestock traceability administrators, such as Pig Trace Canada (*Personal communication: Pascale Aubry, CFIA and Tim Pasma, OMAFRA*). Although CFIA and OMAFRA have a data sharing agreement in the event of an outbreak, access to the livestock location database is restricted to use during an animal disease emergency. Therefore, our objective was to develop a methodology to model farmed swine population in Ontario, Canada that captured the most important aspects of the existing swine farm population structure in Ontario in order to improve the realism of computer simulation models.

## Methods

### Swine farming in Ontario and 2011 census database structure

Statistics Canada conducts an agricultural census once every five years and detailed data for the most recent agricultural census (2011) was publicly available from the Statistics Canada website [3]. The Canadian census data on swine farming consisted of the number and types of farms, and the aggregated number of animals (Additional file 1: Table S1, Figure S1, S2). The smallest unit of the aggregated farming data was Census Consolidated Subdivisions (CCS). Formation of a CCS depended on multiple factors that included a census subdivision consisting of a land area greater than 25 km^2^ or having a human population size greater than 100,000 [12].

Swine farm data were extracted from the 2011 Agricultural Census by CCS, the smallest aggregated geographical unit. However, there were two limitations related to the spatial resolution of the data available for analysis. First, a large proportion of data on the total number of animals in a farm were missing. Based on production type, the missing data ranged from 33% to 48% (Additional file 1: Table S1). And second, the format of the census data did not distinguish mixed farm types (e.g. farms having multiple swine production types), which is a feature of the swine population commonly observed in Southern Ontario [13]. These limitations made the dataset less useful for assessing the overall number of pigs and types of swine farms in each CCS. Therefore, we only utilized the total number of pig farms as reported in the 2011 census for our model. We performed additional data processing to match geographic boundary files of Ontario CCS with the 2011 Census swine population data to develop a geospatial database that matched the swine farm density reported in the 2011 Census data (Additional file 1: Figure S1, S2).

### Data processing and evaluation

We have described the estimation and validation of the spatial locations of swine farms in Ontario, Canada as a six-step process: This included 1) identifying ecological and anthropogenic factors that may affect swine farming; 2) defining the relative importance of the factors affecting swine farming, 3) generating an algorithm to achieve a likelihood score for swine farming in a particular location; 4) estimating the suitability of a location for swine farming; 5) generating random farm locations equal to the swine farm density reported in the 2011 Census of Agriculture, and 6) validate the methods for estimating swine farming likelihood.

### Understanding the factors influencing the locations of swine farms in Ontario

We consulted with swine experts in Ontario (Drs. Zvonimir Poljak and Terri L. O’Sullivan, Ontario Veterinary College, University of Guelph, and Dr. Tim Pasma, Lead Veterinarian and Epidemiologist, Animal Health and Welfare Branch of the Ontario Ministry of Agriculture, Food and Rural Affairs, Guelph, Ontario, Canada) through multiple face-to-face meetings, and email communications, reviewed available farm data, and performed literature searches to determine the factors influencing swine farming in Ontario (Table 1) [14, 15]. Like any other commercial operation, we assumed that access to transportation was one of the key drivers for the establishment of a swine farm. Additionally, we assumed that the local regulations for agricultural land use, land availability and facilities supporting farm infrastructure also played key roles in determining suitable swine farm locations [14, 15]. Key drivers for zoning regulations by the local and provincial authorities included concerns related to water and air pollution and odour control [15]. Production waste (i.e. manure) resulting from intense swine farming was identified as a key environmental concern [15]. Because swine farm waste materials contain a high concentrations of nitrate, phosphate, salts, and bacteria, they may pollute nearby open water sources [15], triggering risks for the health of human, animal, and aquatic ecosystems. As a result, many of the regulations and guidelines focus on containing swine farms within specific agricultural zones in order to have sufficient space to store, handle, and process farm waste materials. Since southern Ontario mostly consists of gently rolling plains, larger waterbodies were the only noticeable natural barriers for the establishment of swine farms [16]. However, because of the current regulations for environmental safety, we assumed that swine farms needed to be situated a reasonable distance from large open waterbodies to prevent contaminants from farm waste materials. Other factors, such as the land designated for population and urban centers, government institutional uses, industrial zones, Crown game preserves, camps and recreational uses, and Crown lands added additional restrictions for areas that could support swine farming in Ontario (Table 1).

**Table 1:** Factors influencing the locations of swine farms in Ontario, scoring guidelines, and geographic information system data used to assign scores

Attributes	Scoring guideline	GIS Data
*Zoning and Local Regulations*		
Population centers	Farms are not allowed to be established within a municipality or a population center	Ontario Population Centers /Residential Land Use
Agricultural zone	Swine farms are encouraged to be established within the area zoned for agricultural use	Agricultural ecumene
Roads within population center	Although farms are almost always near a road network, the roads passing within a population center or residential area should be away from a swine farm	Road Network Passing through a population Center/ Residential Location
*Land Availability*		
Crown Land	Crown Land - MNR Unpatented Land Public are not supposed to have any private establishment. However, since the most recent update in the database (2010), a proportion of the land may have been given access to public. Therefore, we assigned a suitability score of 5% within the Crown Land, and 100% for the remainder	Crown Land - MNR Unpatented Land Public
Crown Game Preserves	Farms are not allowed to be established on land designated for Crown game preserves	Crown Game Preserves
Government Institutional Land Use	Farms are not allowed to be established on land designated for government institutions	Government Institutional Land Use
Industrial Land Use	Farms are not generally allowed to be established on land designated for industrial establishments	Resources and Industrial Land Use
Camp, Recreation	Farms should be located a certain distance from camp and recreational establishments	Camp, Recreation
*Facilities Supporting Farm Infrastructure*		
Road Network	Roads that do not got through population centers have a higher likelihood of being near swine farms	Road Network
	Swine farms could be located near highways, but their access to transportation will depend on the local road network connecting the farms, thus we only kept the local road network attribute.	
*Geographic Barriers*		
Waterbodies	Swine farms are generally not located near shorelines of the large waterbodies (e.g. Great Lakes). Therefore, we considered that it would be unlikely that farms would be within 1 km of the shorelines of the Great Lakes.	Great Lakes
Large Inland Waterbodies	We consider it highly unlikely that a swine farm would be located within the boundaries of large inland waterbodies (e.g. rivers and lakes).	Inland lakes and rivers

### Developing geospatial data layers and assigning suitability scores

To assess the factors influencing swine farming in Ontario (Additional file 1: Figure S3), we identified the geospatial databases that could capture zoning and local regulations, farm land availability, facilities supporting farm infrastructure, and geographic barriers (Table 2). Multiple data processing steps were necessary to assign and combine the suitability scores into a single geospatial dataset. We re-projected all of our geographical data layers to The Lambert Conformal Conic map projection, a map projection system frequently used in Ontario [17].

**Table 2:** Geospatial data layers and the associated suitability scores for the prospect of having a swine farm in a particular geographical location

GIS data layers	Data source	Categories	Suitability scores
Road Network (excluding highways)	StatCan	Roads passing through population centers	0
		Roads that don’t pass through population centers.	
		- Within 300 m	100
		- Between 301 and 500 m	50
		- Between 501 and 5000 m	25
		- More than 5 KM	0
Agricultural Ecumene^a^	StatCan	- Intersect	100
		- Do not intersect	10
Population Centers/Residential Zones	StatCan and DMTI Spatial Inc.	- Within a population center/residential zone	0
		- Within a 2 KM buffer around the outer perimeter of a population center	25
		- Between 2.001 and 15 KM buffer around a population center	95
		- More than 15 KM away from the outer perimeter of a population center	50
Crown Land - MNR Unpatented Land Public	Ontario Land use information, Ministry of Natural resources	- Intersect	5
		- Do not intersect	100
Camp and Recreation spots	Ontario Land use information, Ministry of Natural resources	- Intersect within 1KM buffer of the outer perimeter	0
		- Outside 1KM buffer of the outer perimeter	100
Government Institutional Land Use	DMTI Spatial Inc.	- Intersect	0
		- Do not intersect	100
Resource and Industrial Land Use	DMTI Spatial Inc.	- Intersect	0
		- Do not intersect	100
Waterbodies – Great Lakes	NOAA	- Intersect within 1KM buffer of the outer perimeter	0
		- Outside 1KM buffer of the outer perimeter	100
Large Inland Waterbodies	StatCan	Larger waterbodies (e.g. ranking 1, 2 and 3). A geographical reference location:	
		- Intersect within 1KM buffer of the outer perimeter	0
		- Outside 1KM buffer of the outer perimeter	100

^a^Agricultural Ecumene refers to the occupied land surface used for agricultural or any other economic purposes

We divided the province of Ontario into 1 km^2^ geospatial grids as data units and calculated the distance between each unit and the nearest factors influencing swine farming (Table 2). The next step included assigning suitability scores based on the distance between the outer boundary of a geospatial grid and a factor influencing swine farming (Table 2). For example, the likelihood of a farm being within 300 m of a road that does not intersect a residential location is very high because of the ease of transporting animals and goods. However, as a farm location unit moves further away from road networks, transportation logistics become more difficult. With a conservative approach, we assumed that it would not be logistically feasible to establish a farm five kilometers away from the nearest road network. Similarly, because of the local regulations [15], pig farms could not be established within a population center or residential zone, thus we assigned a likelihood score of zero to these area of the geospatial grid. Initially, we assumed that pig farms would need to maintain a sufficient distance from provincial population centers. However, when we assessed publicly available pig farm location data from Ontario (*n* = 45, data not shown), there were a small proportion of farms within the two kilometer buffer outside the population centers. Therefore, we assigned a lower likelihood score to the geospatial units within the two kilometer buffer outside the population centers than the geospatial units more than two kilometers outside the population centers. We also assumed that the likelihood of a farm being more than 15 km away from the nearest population center is lower because of the logistics and costs associated with animal, animal product, and farm supplies and consumables transportation. Agricultural zones were of specific focus for assigning probability scores because the majority of the surface within the zone consisted of the loam soil types. This particular soil type supports the storage and draining of organic waste materials produced on a farm [14] and for this reason we assigned a high likelihood score for geospatial units within a defined agricultural zone. Because of the current local regulations relating to open water pollution, farms should maintain sufficient distance from open-water sources. Hence, we created a 1-km buffer around the larger waterbodies and calculated the distance between a geospatial unit and the nearest water source. The geospatial units that intersected a water body, received a likelihood score of zero. However, assigning probability scores to the geospatial units near waterbodies presented a challenge because a single geospatial unit may intersect with the 1 km buffer zone, but a large proportion of the surface of the unit might have sufficient space to accommodate a farm (Additional file 1: Figure S4). For this reason, we subtracted the land surface covered by the polygons generated from the ‘1-km buffer to the waterbodies’ from the dataset. Performing this action allowed us to retain portions of a geospatial unit with space that could accommodate a swine farm.

### Combined suitability score for pig farming operations and random farm placement

**Fig. 1:**
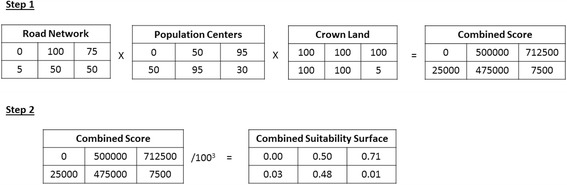
An example of calculating a combined suitability surface: step 1 of the process required multiplying the input parameters with the scores assigned for each of the geospatial units, and step 2, consisted of regressing the combined suitability scores to a scale ranging from 0 to 1

To develop a combined pig farm suitability surface, we developed an algorithm consistent with the suitability scores calculated for each of the individual level factors that were identified as influencing pig farming in Ontario. Figure 1 is an example of calculating a combined suitability surface: step 1 consisted of the process of multiplying the input parameters with scores assigned for each of the geospatial units and step 2 consisted of regressing the combined suitability scores to a scale ranging from zero to one. With zero being completely unsuitable and one being completely suitable based on the defined algorithm.

Once we calculated the combined pig farm placement suitability scores, we generated random farm locations within the geospatial units that exceeded a defined threshold (methods described in the model accuracy assessment section). The random points generated within a CCS were equal to the number of farms reported in the 2011 agricultural census [18]. Additionally, random points were not permitted within 300 m of each other. The minimum distance of 300 m were selected based on the observed distance characteristics of the publicly available pig farm data in Ontario (data not shown).

### Validate the methods for estimating swine farming likelihood

To assess the accuracy of the models, we followed standard approaches to generate agreement matrices [19–21] in order to compare the level of agreement between the predicted combined suitability score of the models and the “reference” presence or absence data. The presence/absence pig farm data for a geographical unit served as a reference dataset for evaluating the classification accuracy of the predictive models.

The reference data on pig farm premises for validation of the model-generated swine population was derived from the Ontario Provincial Premises Registry collected by the Ontario Ministry of Agriculture, Food, and Rural Affairs [22]. The Provincial Premises Registry provides a premises identification number for those who voluntarily registered their agri-food businesses, which includes livestock farms. Although premises registration was voluntary, the compliance rate was very close to 100% because the farms were required to have a premises identification number to report pig movements to meet federal reporting regulation (Personal communication, Ontario Ministry of Agriculture, Food, and Rural Affairs). Most importantly, for the purpose of validating the modeled swine population algorithm, premise registration records included information regarding the geographic location of the centroid of a parcel of land where the farm is located or individual barns of the swine farms. The total number of registered premises within the province of Ontario was approximately double the number of farms listed in the 2011 agricultural census likely because many of the farms registered multiple premises or there was an underreporting of swine farms in the 2011 census or both.

To assess model validity, we compared the algorithm-generated swine farm locations for the modeled swine datasets that received no score (i.e. zero, not suitable for pig farm) with those that received a positive score (i.e. non-zero, could support a pig farm) to the geographical location units that had presence or absence information on pig farm operations from the Provincial Premises Registry. The Provincial Premises Registry served as the reference dataset for model validation.

We developed multiple models by varying the combinations of explanatory variables. To begin with, we identified a minimal required set of explanatory variables that could explain probable pig farming locations and assess the accuracy. Considering the logistical challenges with assigning suitability scores to the surfaces intersecting with waterbodies (Additional file 1: Figure S4) and the probability that farms could have been placed near waterbodies before regulations were in place (e.g. previously described publicly available swine farm location data identified 4.4% of the farms were within one kilometer near a waterbody), we removed the variables explaining different types of waterbodies (from small to large) from models 2 through 5 to assess the changes in model accuracy (Table 3). We initially assumed that the explanatory variables included in model 1 were sufficient to assign a probability score for swine farm locations. However, since the Crown land database (Crown Land - MNR Unpatented Land) was somewhat dated (last update in 2010), we included this variable in model 4 to assess the difference in model accuracy. We also hypothesized that the explanatory variables in model 1 would be sufficient to represent agricultural zones because they excluded the population centers and other non-agricultural related land uses. To explore this, we included the agricultural ecumene to model 6 to assess the changes in model efficacy.

**Table 3:** Accuracy statistics of the model swine populations: area under the receiver operating characteristics curve (AUC) and standard deviations (Sd)

Models	AUC (Sd.)
Model1	0.74 (0.004)
Road network + population centers and residential zones + camp and recreational spots + government institutional land use + resources and industrial land use + great lakes + large inland waterbodies + small inland waterbodies	
Model2	0.75 (0.004)
Removed^a^ small inland waterbodies from *Model1*	
Model3	0.76 (0.004)
Removed large inland waterbodies with 1 KM buffer from *Model2* surface	
Model4	0.76 (0.003)
Included Crown Land - MNR Unpatented public land to *Model3*	
Model5	0.89 (0.003)
Removed great lakes with 1 KM buffer from *Model4* surface	
Model6	0.90 (0.003)
Included *a*gricultural ecumene to *Model5*	

^a^Removed was defined as the section(s) of a surface unit intersecting with a predictor which was not included in the computation

Since we developed multiple models based on a range of different assumptions, we followed a two-step approach for model selection and assessment of validity. First, for each of the population models (Table 3), we calculated the area under the receiver operating characteristic (ROC) curve using the Mann-Whitney U statistic [23]. The hypothesis was that that a randomly chosen 1-km^2^ surface with a pig farm present (from the Provincial Premises Registry) would receive a higher likelihood score compared to the randomly chosen geographical unit without any pig farms. In the second step, True Skill Statistics (measured as: sensitivity + specificity - 1) [24] and Kappa values were calculated to identify an optimal threshold cut-off for the model’s combined suitability score that demonstrated maximum sensitivity and specificity [21, 25].

Once we identified an optimal threshold for the model with the highest area under the ROC curve (AUC), we generated random spatial points from a uniform distribution [18] to represent farm locations for the population model within the geographical surface that scored equal to or above the threshold likelihood score. The number of random modeled farm location points within a Census Consolidated Subdivision (CCS) was equal to the number of pig farms described in the 2011 Agricultural Census. Once the farm locations were assigned, we randomly assigned the type of swine operation (e.g. farrow to finish, finisher farm types) proportional to the recently surveyed operation types [13]. The farm types were also randomly assigned a size (number of pigs per farm) based on the farm-level population distribution from the 2011 census information.

## Results

**Fig. 2:**
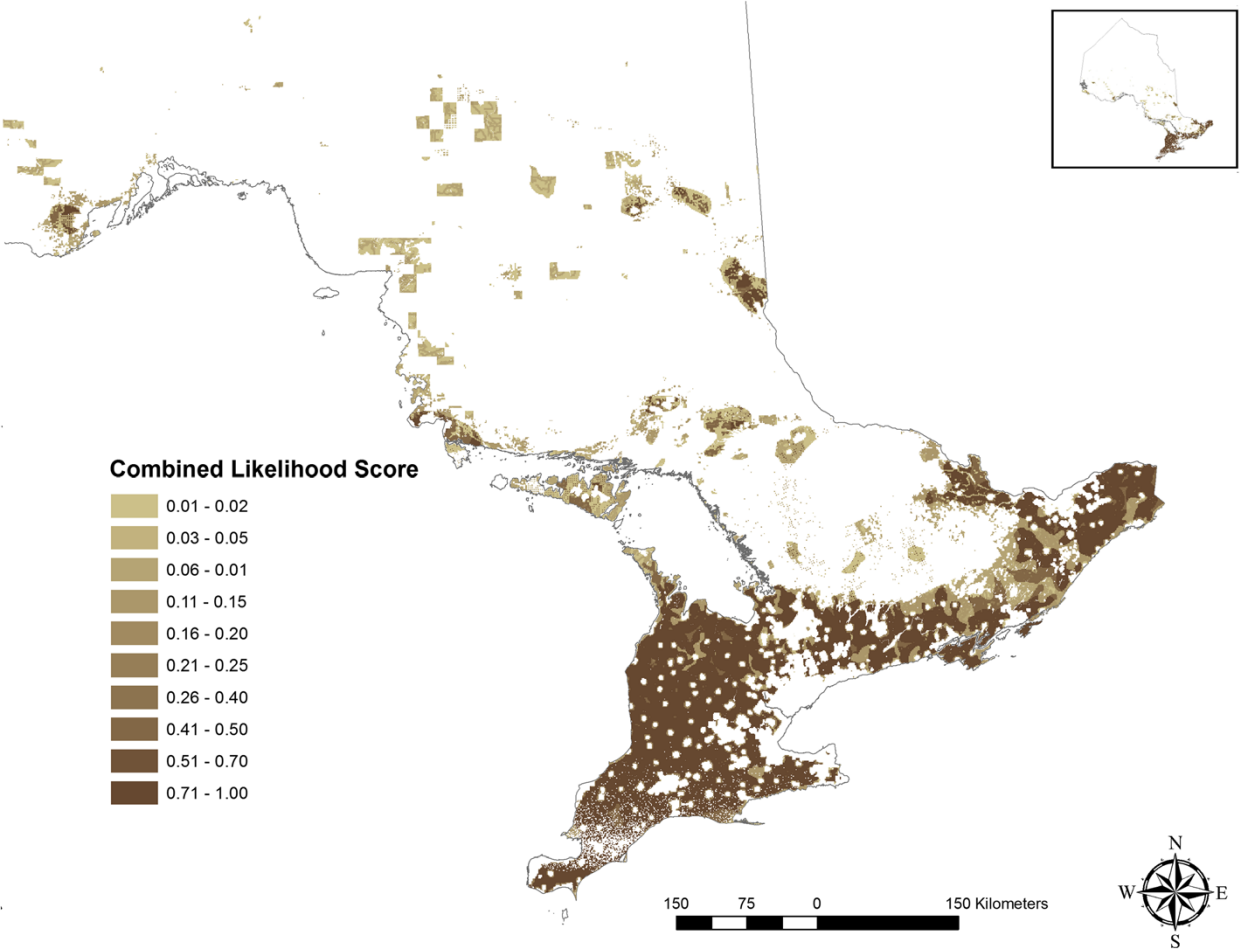
A map of Ontario showing the combined pig farming likelihood scores generated through model 6. The intensity of the color represents the combined likelihood score (range 0–1): the areas with a higher likelihood score (darker color) represent a greater probability of having a pig farm and the surfaces in white represent areas with no probability of having a pig farm

**Fig. 3:**
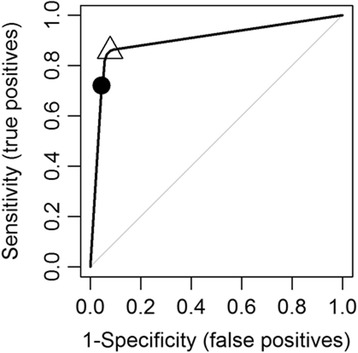
A receiver operating characteristic (ROC) plot for the combined likelihood score’s predictability of model 6, with the optimal threshold marked along the ROC curve. The hollow triangle shape and the black circle indicate the sensitivity and specificity associated with True Skill Statistics (0.04) and the maximum Kappa value (0.82) respectively

**Fig. 4:**
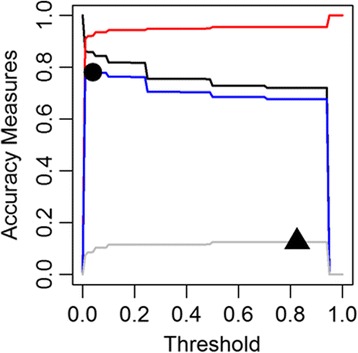
A graph of error measures (sensitivity, specificity, True Skill Statistics and Kappa) as a function of the threshold for model 6. The colored lines representing sensitivity (black), specificity (red), True Skill Statistics (blue), and Kappa (gray). The black circle indicates the threshold cut-off (0.04) where True Skill Statistics was highest and the triangle shape indicates the threshold cut-off (0.82) where maximum Kappa value was achieved

**Fig. 5:**
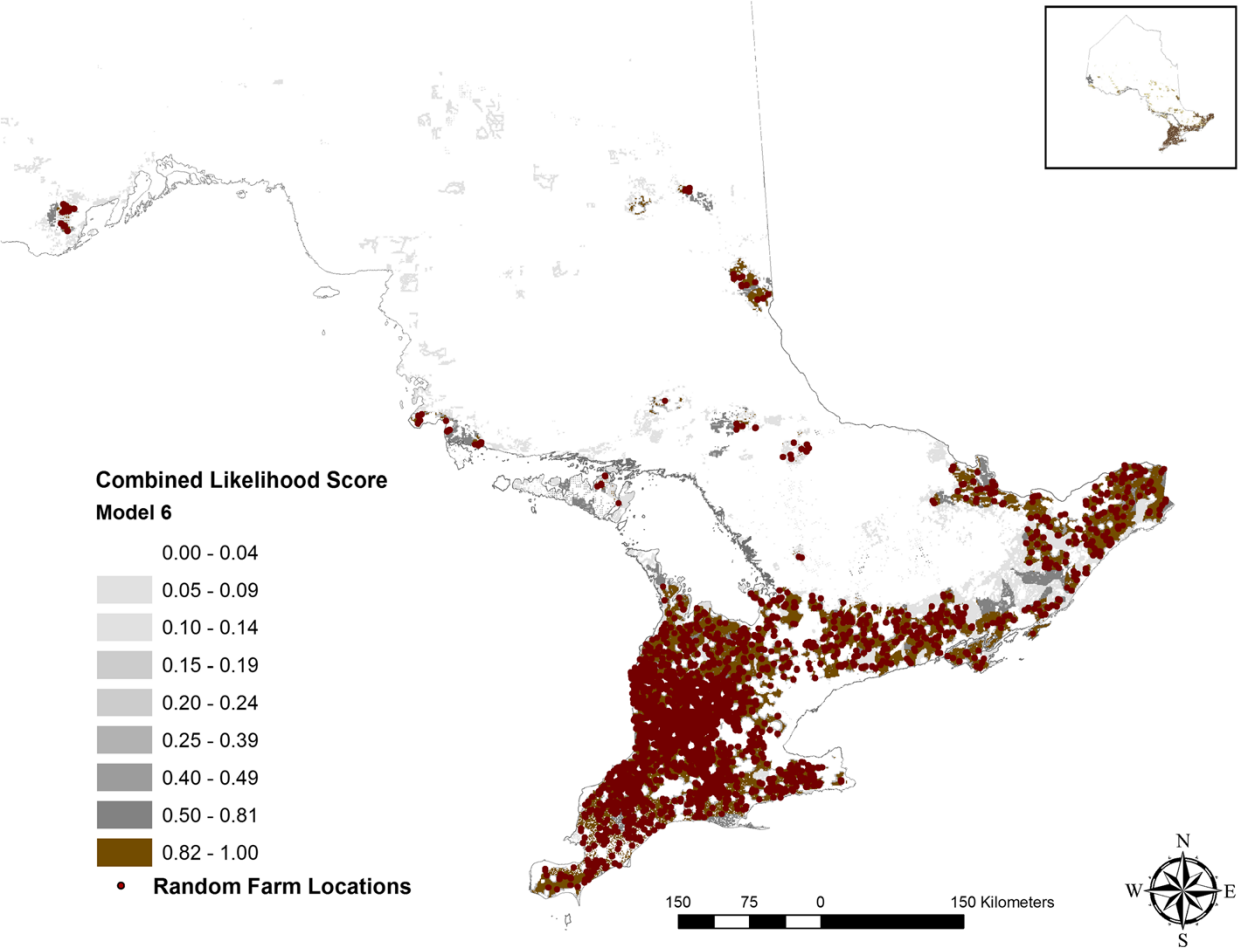
Random pig farm locations generated using model 6 and based on a combined likelihood threshold cut-off value of 0.82.The red dots (*n* = 2485) represent the locations of model pig farms generated on the surfaces that fall above the likelihood threshold cut-off value. The color intensity on the background represents the combined likelihood threshold

Attributes that received a suitability score to support swine farming had varying degrees of land surface coverage: highest land surface (31%) was covered by the proximity to the road network and the least (0.6%) was by government and institutional land use (Additional file 1: Table S2). Models to generate a combined likelihood scores to identify pig farming locations in Ontario demonstrated relatively high accuracy (Table 3). A suitable model (Model 6, Fig. 2) was achieved by incorporating distance information on road networks, population centers and residential zones, camps and recreational centers, land used by government, resources, and industrial purposes, and agricultural zoning and subtracting the landscape areas that had larger waterbodies or were within a 1 km buffer zone of a larger waterbody. Model 6 demonstrated a very high predictive accuracy (AUC: 0.90, Standard deviation (SD): 0.003) (Fig. 3) when compared to the premises registry information describing pig farming in Ontario (Table 3). The model predicted 9.6% of the Ontario’s land surfaces, mostly in southern Ontario suitable for supporting pig farms. When comparing model algorithms to the true pig farming locations (premises registry), we used the higher threshold cut-off identified between two distinct approaches: True Skill Statistics and Kappa Statistics. A likelihood threshold of 0.82 was identified as an optimal cut-off (Sensitivity: 72%, Specificity: 96%) (Figs. 3 and 4). The land surfaces that scored on and above this threshold cut-off were selected to generate random farm locations (*n* = 2485) equal to the number of swine farms documented in the 2011 Canadian agricultural census. When compared to the known pig farming locations (census consolidated subdivisions) described in the 2011 census, the randomly generated farm location points (Fig. 5) demonstrated very high accuracy (AUC: 0.93, SD: 0.01).

## Discussion

We developed a model to generate a swine population to represent the farm population structure for commercially raised pigs in Ontario. Publicly available data from the 2011 Canadian agricultural census and geospatial information were used to generate a swine population structure that represents an improvement over alternative methods, such as the random placement of farm locations across the landscape [26]. When compared with the actual geospatial location information for pig farms in Ontario, the model demonstrated high accuracy. Generating a well validated model population of swine farms means that this model population could be used as an input into disease simulation models. This will enable researchers to examine questions related to the prevention and control of pathogens within the swine industry including FAD, while at the same time protecting the privacy of individual pork producers. This approach could be replicated for other agricultural commodity groups (e.g. poultry, beef cattle etc.) for which limited individual level data exist.

Our approach was to identify a minimum set of predictors required to assign each cell of a regular grid applied across the geographical extent of Ontario a score reflecting the probability that a pig farm would be present. We hypothesized that regional, cultural, regulation, and landscape factors would be major drivers of the establishment of swine farms. We assigned suitability scores based on a combination of expert opinion, literature reviews, and logic. The models developed based on different combinations of predictors have demonstrated moderate to high accuracy (Table 3). The combination of predictors to create multiple models further helped to assess the predictor selection procedure, particularly the importance of incorporating ‘logic’ (step 6) to improve model accuracy. In addition, we matched the production types and number of animals on each farm based on the known distribution from recent surveys [13] and the 2011 census [3] to develop a realistic population structure for the province of Ontario. Our findings provide a methodology to estimate the likely geographic location of swine enterprises in a given area. This approach can be used to simulate a population of farms that provides a reasonable estimate of both the number of farms and the location of farms in a study area. A similar approach has been used to model commercial poultry farm locations in the United States [26]. The modeled poultry population was later used to generate parameter estimates for highly pathogenic avian influenza transmission models [26, 27].

There has been tremendous growth in the Canadian swine industry over the past few decades. However, profits have varied dramatically for several reasons, including the increased input costs (e.g. feed), disease outbreaks, changes in the value of the Canadian dollar, and changes in North American pork prices [28]. FAD outbreaks are particularly important not only because of the direct impact on production, but also because of the destruction of healthy animals due to welfare slaughter, import bans by Canadian trading partners, and the risk of a long-term import ban by some countries [2, 3]. Between 1921 and 2011, agricultural intensification in commercial swine production has decreased the number of Canadian swine farms from 711,090 to 7371, but in the same time period, the number of pigs raised has increased from 9 million to 33 million pigs [29]. Although the transformation from backyard swine farms to commercial farming operations increases the likelihood that good biosecurity practices will be carried out, the potential introduction of a FAD remains a major concern for the Canadian swine industry. One recent example was in 2009, when countries such as China and Russia placed a ban on Canadian pork imports because of pandemic influenza A (H1N1). This forced some producers to downsize or exit the industry through government assistance programs [1, 30].

The introduction of a FAD into the Canadian swine industry would have an enormous economic impact. In order to mitigate this risk, preparedness planning is required to estimate the possible extent of an FAD outbreak, the expected efficacy of the control and eradication measures that would be applied in the event of an outbreak, and the human resource and infrastructure requirements (e.g. the number of veterinarians, technicians, disposal services etc.) necessary to effectively carry out control and eradication measures. One of the key requirements for preparedness planning requires the development and parameterization of disease transmission models that incorporate accurate farm population structure that would serve as the base for the disease transmission model [10, 11]. The methodology described here, provides one such example of how this population structure can be derived. The spread of a livestock pathogen is primarily influenced by three factors: first, the variability of disease susceptibility in different production types, second, the geographical proximity and clustering of farms, and third, the direct and indirect movement of animals, people, feed, fomites, vehicles and animal products between different production types [31–34]. Estimating the direct and indirect movements between farms was out of the scope of this research however, to capture the static swine farm characteristics, we developed a spatially explicit population of swine farm locations in Ontario, classified the farms to different production types based on a recent survey [13], and assigned numbers of animals in each farm based on the farm-level population distribution from 2011 agricultural census. The farm locations were randomly generated across the land surface with a likelihood of supporting a swine farm and farm density matched with the census units. The production types and the numbers of animals in each farm were assigned based on surveys, reports, and census data in order to best replicate the existing swine population in the modeled population [3, 13]. The accuracy statistics demonstrated the geographical locations generated for random farm placement using our model algorithm had a very high agreement with the actual observed swine farm locations and therefore, the model swine population generated is a suitable surrogate for incorporation into disease transmission models.

Although the modeled population was a suitable representation of the actual swine farm locations in Ontario, this is not without limitations. First, the number of farms generated in the models were based on the 2011 agricultural census. Underreporting of swine farms, particularly small and backyard farms in the census could have resulted in generating fewer farms in our model. Moreover, a farm with modest number of pigs (e.g. ~ 700 heads) could have multiple barns located in geographically dispersed location and it was unclear if the census recorded them as a single farm or counted them separately (Personal communication: Dr. Zvonimir Poljak, Department of Population Medicine, Ontario Veterinary College, University of Guelph, Guelph, ON, Canada). On the other hand, the premises registry listed almost twice the number of farm premises. This is likely because individual farm owners had the option to register multiple barns of a single “farm” separately. However, because of the restricted nature of premise registry data, we were unable to verify the proportion of farms that may have registered multiple barns. One way to overcome this issue would be to include a ‘correction factor’ to adjust the estimated number of farms so it better matches the number of farms derived from a different farmed animal data source(s). However, introducing a ‘correction factor’ would require a thorough understanding of the proportion of missing data from the primary data source, in our case the 2011 agricultural census. Since we were unable to identify a credible data source for the proportion of farms missing in the census data, we did not include a ‘correction factor’ with an assumption that the proportional density of the swine farms in the model would be about the same as the actual population. Second, we defined a threshold cut-off using Kappa statistics. This measure is dependent on the prevalence of an outcome [24] and could result in poor agreement when the prevalence is low. In our case, the number of 1-km^2^ grid cells having swine farms was very low compared to the total land surface of the province (0.028 vs. 1). A way to overcome this low-prevalence issue would be to calculate True Skill Statistics. However, the threshold defined by True Skill Statistics was extremely low (Fig. 4). Therefore, we took a conservative approach and selected the threshold cut-off where the Kappa value minimized commission error (higher specificity). Third, we did not incorporate the waterbodies that had extremely small surface areas. The reason for such consideration was because our measurement units were 1 km^2^ geographic blocks. Even if a unit contained a small waterbody, it would have had an adequate amount of surface areas remaining to also accommodate a swine farm (Additional file 1: Figure S4). Finally, we did not incorporate a separate geospatial data layer on soil types in the models. Although loam type soil facilitates farm waste storage, the incorporation of an agricultural ecumene data layer that included much of the land surface with supported soil types as well as easy access to local grain production for swine feeding management was considered to be a reasonable substitute.

## Conclusions

In the absence of a full, identifiable population structure dataset for swine farms in Ontario, developing a model swine population that captures the key characteristics of the observed population structure while protecting producer privacy is an important methodological advancement. Such a population structure will be useful for individuals interested in modeling the spread of pathogens between farms across a landscape and using models to evaluate disease control and eradication measures.

## Additional file

Additional file 1: Modeling Livestock Population Structure: A Geospatial Database for Ontario Swine Farms. Description of data: **Table S1.** Data structure and missing data in 2011 agriculture census: pig farm types and number of heads. **Figure S1.** Frequency of pig farms in Ontario’s census consolidated subdivisions: Canadian Agricultural Census 2011. **Figure S2.** Frequency of pigs in Ontario’s census consolidated subdivisions: Canadian Agricultural Census 2011. **Figure S3.** A map of Canada showing the administrative boundaries of the provinces. **Figure S4.** An example of relative occupancies of small waterbodies in the 1-km^2^ spatial grids. **Table S2.** Land surface covered by the attributes with a suitability score (above zero) supporting swine farms in Ontario. (PDF 336 kb)

## Abbreviations

AUC: Area Under the Receiver Operating Characteristics Curve
CCS: Census Consolidated Subdivisions
CIFA: Canadian Food Inspection Agency
FAD: Foreign animal diseases
GIS: Geographical Information System
OMAFRA: Ontario Ministry of Agriculture, Food and Rural Affairs
ROC: Receiver Operating Characteristics
SD: Standard deviation
