# Supplementary material for: Modeling livestock population structure: a geospatial database for Ontario swine farms
Source: BMC Vet Res. 2018 Jan 30;14:31. doi: 10.1186/s12917-018-1362-y (PMC5791355; doi:10.1186/s12917-018-1362-y)
Supplement: Additional file 1: — Modeling Livestock Population Structure: A Geospatial Database for Ontario Swine Farms. Description of data: Table S1. Data structure and missing data in 2011 agriculture census: pig farm types and number of heads. Figure S1. Frequency of pig farms in Ontario’s census consolidated subdivisions: Canadian Agricultural Census 2011. Figure S2. Frequency of pigs in Ontario’s census consolidated subdivisions: Canadian Agricultural Census 2011. Figure S3. A map of Canada showing the administrative boundaries of the provinces. Figure S4. An example of relative occupancies of small waterbodies in the 1-km2 spatial grids. Table S2. Land surface covered by the attributes with a suitability score (above zero) supporting swine farms in Ontario. (PDF 336 kb) [file 12917_2018_1362_MOESM1_ESM.pdf]

ADDITIONAL FILE 1

Modeling Livestock Population Structure: A Geospatial Database for Ontario Swine Farms

Salah Uddin Khan*[a], Terri L. O'Sullivan[a], Zvonimir Poljak[a], Janet Alsop[b], Amy L. Greer*[a]

[a]Department of Population Medicine, Ontario Veterinary College, University of Guelph

[b]Ontario Ministry of Agriculture, Food and Rural Affairs

*sukhanbd@uoguelph.ca, agreer@uoguelph.ca

LIST OF CONTENTS:

1. Table A.1. Data structure and missing data in 2011 agriculture census: pig farm types and number of heads
2. Figure A.1. Frequency of pig farms in Ontario's census consolidated subdivisions: Canadian Agricultural Census 2011
3. Figure A.2. Frequency of pigs in Ontario's census consolidated subdivisions: Canadian Agricultural Census 2011
4. Figure A.3. A map of Canada showing the administrative boundaries of the provinces
5. Figure A.4. An example of relative occupancies of small waterbodies in the 1-square kilometer spatial grids

6. Table A.2. Land surface covered by the attributes with a suitability score (above zero) supporting swine farms in Ontario

Table A.1: Data structure and missing data in 2011 agriculture census: Number and percentage of Census Consolidated Subdivision (CCS) with missing information on pig farm types and number of heads

Units (N=273 CCS)	Missing (%)
Total number of pig farms	0 (0%)
Total number of pigs	129 (47%)
Total number of boar farms	0 (0%)
Total number of boars	107 (39%)
Total number of saw and gilt farms	0 (0%)
Total number of saws and gilts	121 (44%)
Total number of nursing and weaner farms	0 (0%)
Total number of nursing and weaners	113 (41%)
Total number of nursing farms	0 (0%)
Total number of nursing pigs	89 (33%)
Total number of weaner farms	0 (0%)
Total number of weaners	116 (43%)
Total number of grower and finisher farms	0 (0%)
Total number of growers and finishers	132 (48%)

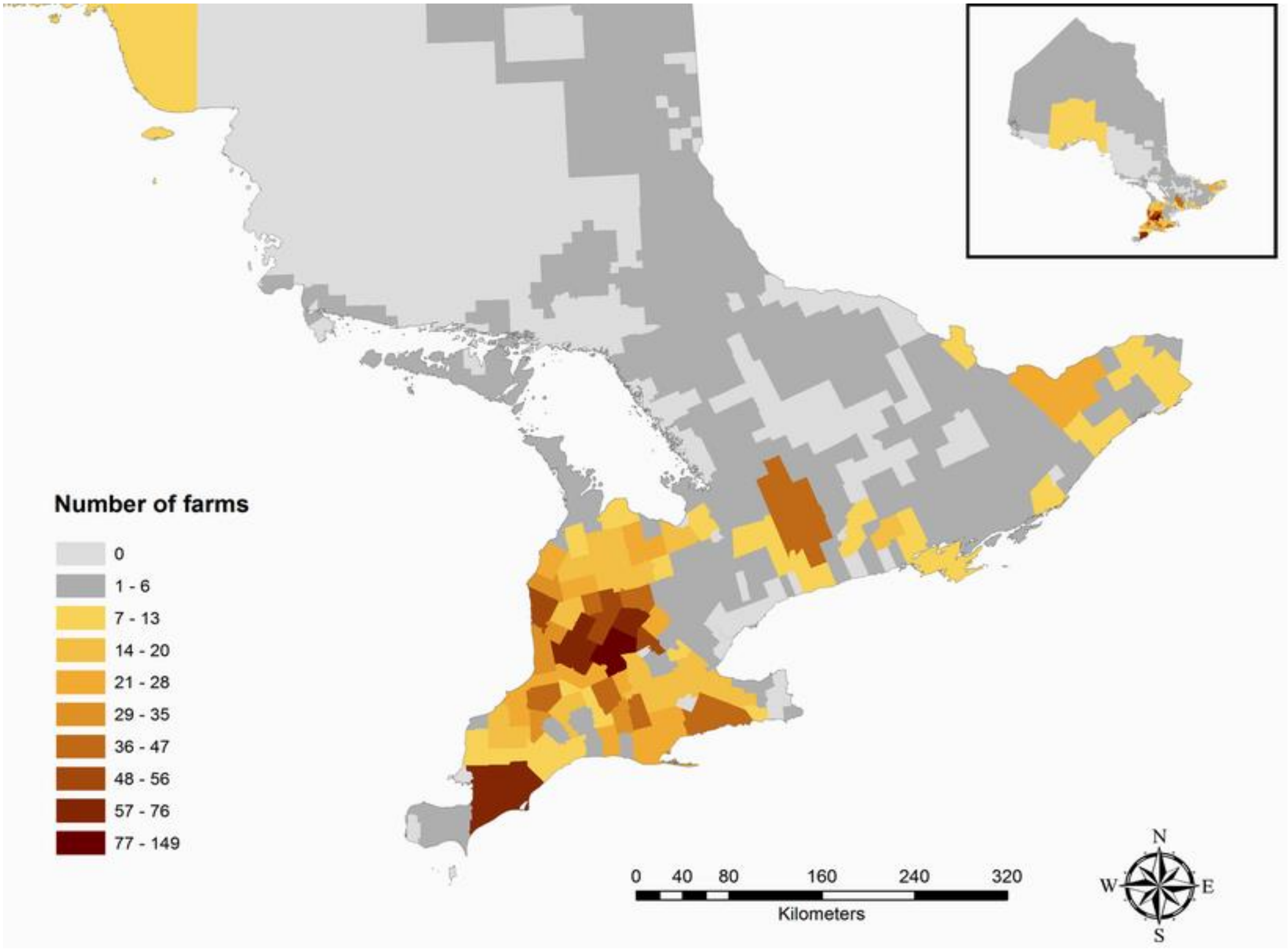

Figure A.1. Number of pig farms in each census consolidated subdivisions: Canadian Agricultural Census 2011.

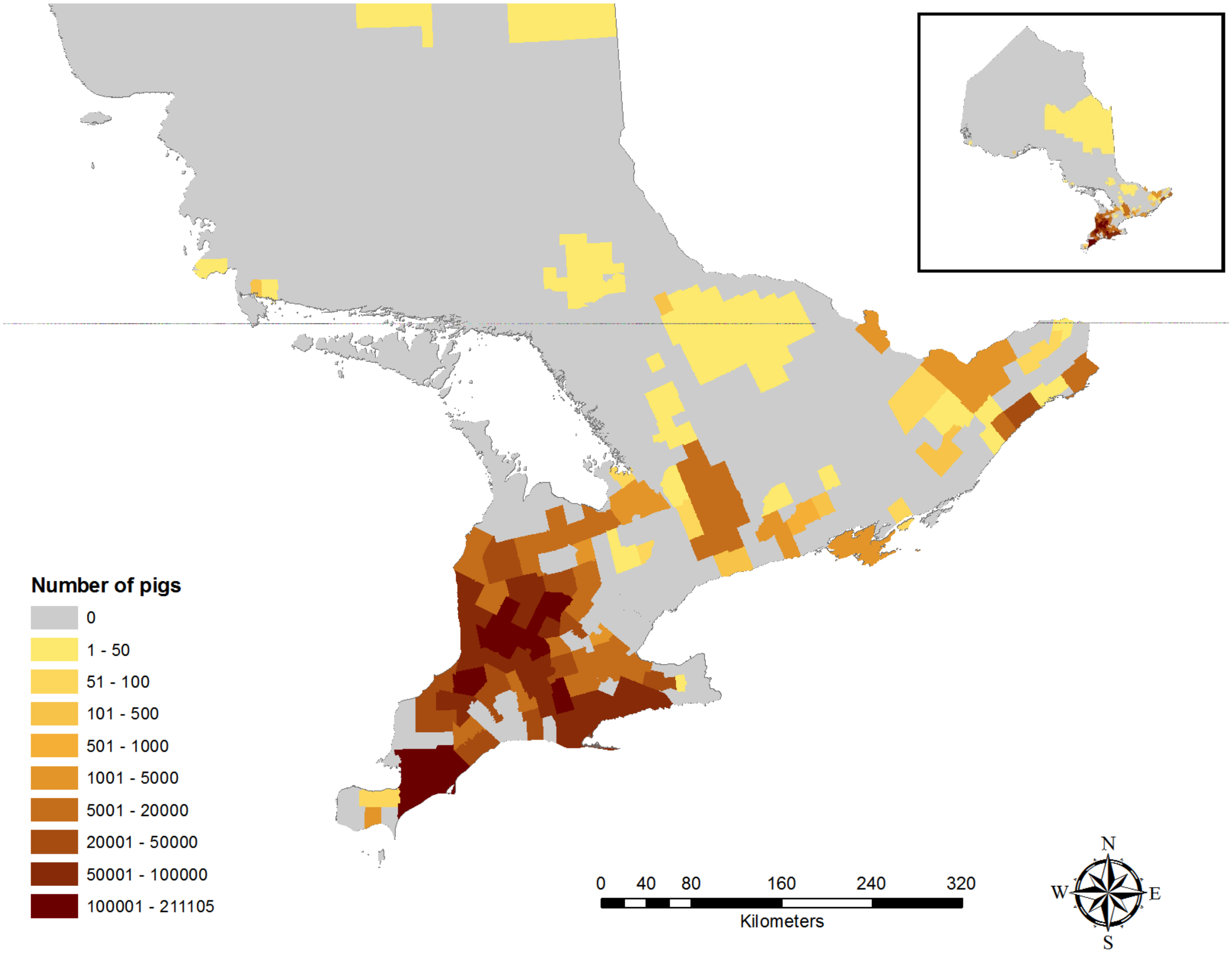

Figure A.2. Number of pigs in each of Ontario's census consolidated subdivisions: Canadian Agricultural Census 2011.

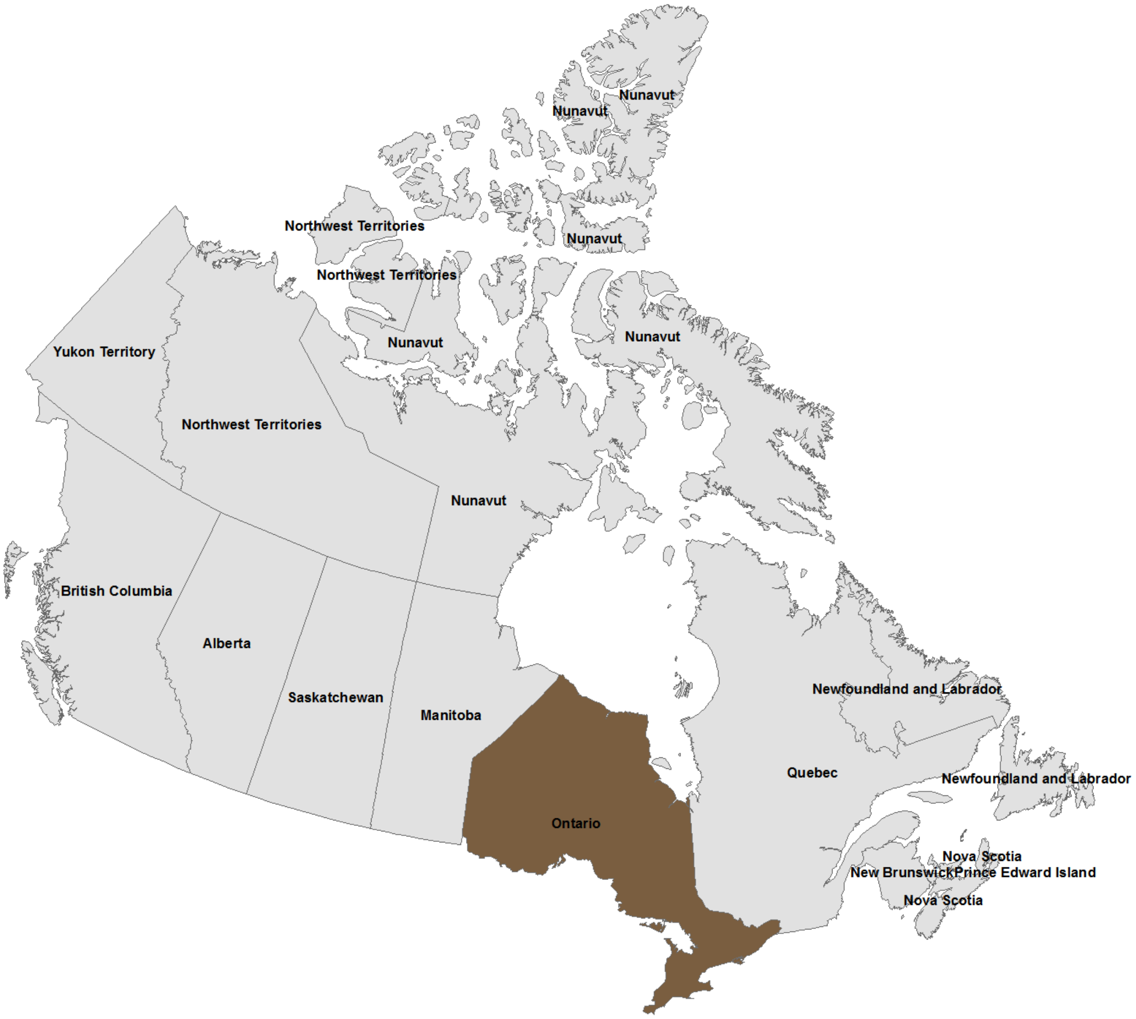

Figure A.3. A map of Canada showing the administrative boundaries of the provinces.

The province of Ontario is shaded.

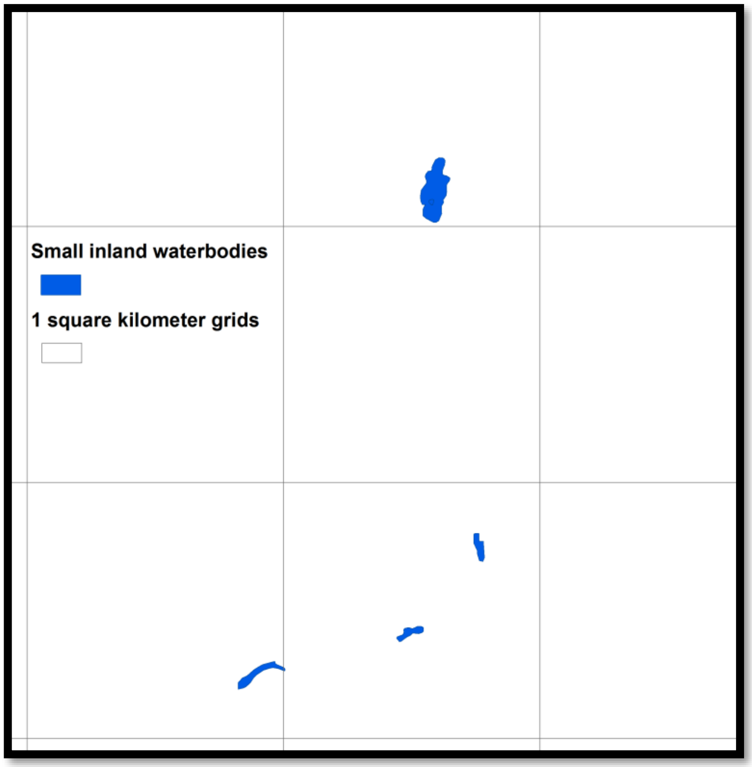

Figure A.4. An example of relative occupancies of small waterbodies in the 1 square kilometer spatial grids leaving enough room for swine farm establishments within a cell containing a small waterbody or small proportion of a larger waterbody. The grey lines demarked the boundary of a one square kilometer cell used as a unit for assigning suitability scores and the blue colored polygons represented the relative positions of waterbodies within the cells.

Table A.2. Land surface covered by GIS data layers of attribute's suitability score supporting swine farms in Ontario

**Attribute / GIS Data Layer**	**Categories**	**Suitability Score**	**Land Surface Covered (%)**
Road Network (excluding highways)	Roads that don't pass through population centers.		
	- Within 300 meters	100	20.2%
	- Between 301 and 500 meters	50	1.9%
	- Between 501 and 5000 meters	25	8.8%
	- More than 5 KM	0	69.1%
Agricultural Ecumene	- Intersect	100	8.4%
	- Do not intersect	10	91.6%
Population Centers/Residential Zones	- Within a population center/residential zone	0	1.1%
	- Within a 2 KM buffer around the outer perimeter of a population center	25	1.4%
	- Between 2.001 and 15 KM buffer around a population center	95	9.8%
	- More than 15 KM away from the outer perimeter of a population center	50	87.7%
Crown Land - MNR Unpatented Land Public	- Intersect	5	88.8%
	- Do not intersect	100	11.2%
Camp and Recreation spots	- Intersect within 1KM buffer of the outer perimeter	0	1.4%
	- Outside 1KM buffer of the outer perimeter	100	98.6%
Government Institutional Land Use	- Intersect	0	0.6%
	- Do not intersect	100	99.4%
Resource and Industrial Land Use	- Intersect	0	0.8%
	- Do not intersect	100	99.2%
Waterbodies – Great Lakes	- Intersect within 1KM buffer of the outer perimeter	0	3.6%
	- Outside 1KM buffer of the outer perimeter	100	96.4%
Large Inland Waterbodies	Larger waterbodies (e.g. ranking 1, 2 and 3). A geographical reference location:	0	10.6%
	- Intersect within 1KM buffer of the outer perimeter	100	89.4%
	- Outside 1KM buffer of the outer perimeter		
